# Assessing hearing by measuring heartbeat: The effect of sound level

**DOI:** 10.1371/journal.pone.0212940

**Published:** 2019-02-28

**Authors:** Mehrnaz Shoushtarian, Stefan Weder, Hamish Innes-Brown, Colette M. McKay

**Affiliations:** 1 The Bionics Institute, East Melbourne, Victoria, Australia; 2 Department of ENT, Head and Neck Surgery, Inselspital, Bern University Hospital, Bern, Switzerland; 3 The University of Melbourne, Department of Medical Bionics, Melbourne, Australia; University College London, UNITED KINGDOM

## Abstract

Functional near-infrared spectroscopy (fNIRS) is a non-invasive brain imaging technique that measures changes in oxygenated and de-oxygenated hemoglobin concentration and can provide a measure of brain activity. In addition to neural activity, fNIRS signals contain components that can be used to extract physiological information such as cardiac measures. Previous studies have shown changes in cardiac activity in response to different sounds. This study investigated whether cardiac responses collected using fNIRS differ for different loudness of sounds. fNIRS data were collected from 28 normal hearing participants. Cardiac response measures evoked by broadband, amplitude-modulated sounds were extracted for four sound intensities ranging from near-threshold to comfortably loud levels (15, 40, 65 and 90 dB Sound Pressure Level (SPL)). Following onset of the noise stimulus, heart rate initially decreased for sounds of 15 and 40 dB SPL, reaching a significantly lower rate at 15 dB SPL. For sounds at 65 and 90 dB SPL, increases in heart rate were seen. To quantify the timing of significant changes, inter-beat intervals were assessed. For sounds at 40 dB SPL, an immediate significant change in the first two inter-beat intervals following sound onset was found. At other levels, the most significant change appeared later (beats 3 to 5 following sound onset). In conclusion, changes in heart rate were associated with the level of sound with a clear difference in response to near-threshold sounds compared to comfortably loud sounds. These findings may be used alone or in conjunction with other measures such as fNIRS brain activity for evaluation of hearing ability.

## Introduction

Functional near-infrared spectroscopy (fNIRS) is a non-invasive brain imaging technique that measures changes in oxygenated and de-oxygenated hemoglobin concentration (HbO and HbR). fNIRS provides a measure of brain activity since neural activation increases metabolic demand, leading to increased blood flow and changes in HbO and HbR concentration levels. In addition to neural activity, raw fNIRS signals contain systemic oscillations that are related to physiological processes such as heartbeat [1–3]. Although these oscillations are often removed in the processing of fNIRS data, they can be used to extract useful information regarding cardiac dynamics. This paper investigated whether changes in cardiac signals extracted from fNIRS data are associated with level of sound. If so, the cardiac information could potentially be used on its own, or combined with other objective measures of hearing, to determine the hearing range of listeners (i.e. the sound levels that evoke threshold perception and comfortably loud sensations). fNIRS is well-suited as an imaging technique for hearing research as it is non-invasive, portable and functions silently (compared for example to functional magnetic resonance imaging or fMRI). In recent years a number of studies have used fNIRS to investigate cortical processing of different sounds and sound features (e.g. [4–6]).

Several studies have looked at the effect of different sound features on heart rate and heart rate variability. In newborns band-limited noise at moderate levels was shown to elicit a reliable increase in heart rate [7]. Schulman reported that this response did not increase significantly with increasing sound [8] however, the stimulus range used was limited to close to the behavioral threshold level. A number of earlier studies also investigated changes in heart rate with tones at different frequencies and durations [9–14]. Changes in heart rate found in these studies were interpreted as autonomic responses to stimulation. These and similar studies [15], however, focused on either single sound levels or multiple levels at moderate and loud intensities. In this experiment we applied a range of intensities from near-threshold to louder levels to investigate whether changes in heart rate were associated with these sound levels. These findings could potentially be used to deduce an individual’s hearing range between threshold level and comfortably loud level. This information could have important clinical utility in objectively assessing hearing and for objective hearing device programming in hearing impaired infants or adults who are unable to perform behavioral hearing assessments.

We have previously shown that fNIRS brain-activity responses are dependent on the intensity of auditory stimuli [5]. In this paper we have instead analyzed the raw fNIRS data to extract cardiac related information. We aimed to investigate whether changes in cardiac activity in response to four sound levels were dependent on the intensity of the sound. If successful, the results could be used to develop methods to combine the information from both cardiac activity and neural activity to increase the efficacy of fNIRS in objective hearing assessments.

## Materials and methods

### Participants

Twenty-eight healthy adults were recruited (average age: 30 (range 24–39), 12 females). One participant was excluded due to a reported heart condition. Participants had no history of neurological or hearing disorders. Pure tone audiometry performed on the same day as fNIRS data collection showed that hearing in all participants was within the normal range (hearing threshold less than 20dB Hearing Level (HL) at frequencies 125 to 8000 Hz).

The study was approved by the Royal Victorian Eye and Ear Hospital Human Research Ethics Committee (project number 16/1261H). Written informed consent was obtained from all participants.

### fNIRS recordings

A multi-channel continuous-wave fNIRS system operating at 760 and 850 nm (NIRScout, NIRx Medical Technologies LLC) was used to collect data. A total of 16 sources and 16 detectors were placed over the temporal and frontal scalp. Source-detector pairs were placed 3cm apart, forming 32 channels. In this study, data from all channels was used to extract heart rate as described further below.

### Acoustic stimulation

Auditory stimuli were delivered binaurally via audiometric insert earphones (ER-3A insert earphone, E-A-RTONE^TM^ 165 GOLD, USA). Stimuli consisted of 18-second segments of ICRA noise (International Collegium of Rehabilitative Audiology) [16] which consists of signals which share similar spectral and temporal characteristics with speech, but are not understandable as speech. Five different segments of the ICRA noise were randomly applied during testing. We used this broadband stimulus to strongly activate cortical auditory areas and reduce habituation due to repetitions of stimuli (see Weder et al. [5] for further details).

### Experimental design

fNIRS testing was performed in a sound-treated booth. Participants sat on a comfortable chair and auditory stimuli were presented using Presentation software (Neurobehavioral Systems, USA). ICRA noise stimuli of 4 different intensity levels (15, 40, 65 and 90 dB SPL) were presented in a counter-balanced block design. Thresholds for the ICRA noise stimuli were obtained using an adaptive three-alternative forced-choice method [17]. Stimuli at 90 dB SPL were subjectively rated by participants as ‘comfortably loud’ or ‘loud but tolerable’.

**Fig 1:**
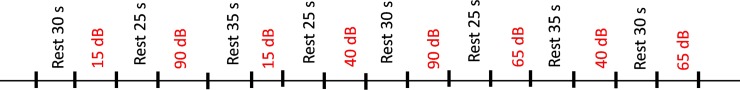
Block design of fNIRS experiment. Stimuli and rest times in an example test period are shown. Eighteen-second duration stimuli were presented twice at each level in counterbalanced order. Rest periods were 25, 30 or 35 seconds (chosen at random). One recording session consisted of 5 test periods with breaks of 2–3 minutes in between.

The fNIRS test session consisted of 5 test periods lasting 7 minutes each, with breaks given between test periods (Fig 1). Data was recorded at a sampling rate of 7.8125 Hz. Each test period started with a 30 second resting period. In each test period, each sound level was repeated twice in a counter balanced order (resulting in 8 stimulus blocks in total). Each stimulus block lasted 18 seconds, followed by a rest period of 25, 30 or 35 seconds chosen at random. In total, stimuli at each level were repeated 10 times (twice in each test period) and the total recording time (without breaks) was 35 minutes. Participants were instructed to listen to the stimuli and to press a button at the end of the sound (in order to maintain their attention).

### Heart rate extraction from fNIRS signals

Data processing was performed in Matlab 2016b (Mathworks, USA). Homer2 functions [18] and custom written Matlab scripts were used for pre-processing of fNIRS signals. All filtering was done using Homer2 functions [18]. In order to select channels with good signal quality and clear heart rate components, two criteria were used. First, channels with gains over 7, which showed inadequate detected light intensity, were rejected from further analysis. To calculate gain, a calibration procedure is performed by the fNIRS system prior to each measurement. In this step the instrument determines the optimum gain (or signal amplification) for each source-detector combination. In addition, it is known that a clear cardiac signal in fNIRS signals is indicative of good contact between optodes and the scalp [2, 19]. Therefore, the scalp coupling index (SCI), was determined [19]. To calculate SCI, the two detected signals at 760 and 850 nm wavelengths were band-pass filtered between 0.5 to 2.5 Hz and then correlated. If the optodes are well-coupled to the skin, the signal in the filtered range should mainly include heart rate data, and the two channels should therefore be highly correlated. Channels with an SCI less than 0.75 were rejected.

For the remaining channels, the original (unfiltered) data was converted to optical density and motion artefacts corrected using Homer2 function *hmrMotionCorrectWavelet (function parameter set to 1*.*5)* which uses wavelet coefficient distributions to remove artefacts [18, 20]. Signals were then band-pass filtered between 0.5 and 1.5 Hz (corresponding to heart rates between 30 and 90 beats per minute). For each channel, the heart rate was then extracted from the 760 nm wavelength optical density signals based on the method described by Purdue et al. [1]. Briefly, heart rate and inter-beat intervals were calculated for each channel, epoched around time of stimulus and averaged across channels and the 10 repeated trials. Details are described below.

Data was up-sampled to 100 Hz to enable better estimation of inter-peak intervals. Upsampling was performed using Matlab’s *resample* function which uses interpolation and an anti-aliasing filter to resample the signal to the desired frequency. To ensure peaks found corresponded to heartbeats and were not signal artefacts, inter-peak intervals and peak widths (calculated using Matlab’s *findpeaks* function) were checked. Peaks were rejected if the width was larger than the mean + 1.5 SD of total averaged widths or IPIs were greater than the mean + 2 SD of averaged IPIs (assumed dropped beats) [1]. From the remaining peaks, two measures were derived, inter-beat intervals and heart rate. Inter-beat intervals were used to quantify the time at which changes in heart rate first occurred. Inter-beat intervals were calculated as the time between successive peaks. The inverse of the inter-peak intervals multiplied by 60 gave a measure of heart beats per minute or heart rate. Heart rate data was then down-sampled to 20Hz (to generate samples at equi-distant time points for later averaging) and low pass filtered at 0.3Hz. Heart rate was calculated in this way for each channel. Channels with heart rate values outside the mean heart rate across all channels plus/ minus 20 beats per minute were rejected. For each remaining channel, heart rate and inter-beat intervals were epoched from t = -5 to t = 30 seconds relative to stimulus onset and averaged across channels. For both measures, the percentage change relative to baseline (defined as heart rate averaged over five seconds before stimulus onset and for inter-beat intervals, 5 averaged pre-stimulus intervals), was calculated. This was done for all time points in the epochs resulting in pre-stimulus values around 0. To quantify the variability of resting heart rate, a similar ‘epoch’ was created with resting data recorded at the start of each recording (see S1 Appendix and S1 Fig).

Percentage change in heart rate and inter-beat intervals compared to pre-stimulus baseline at different sound intensity levels were compared using mixed linear models with subject treated as a random factor and time (pre and post stimulus) and intensity levels treated as fixed factors. Pre-stimulus measures were calculated as mean heart rate across 5 seconds before stimulus onset and 5 averaged pre-stimulus inter-beat intervals. Post-stimulus heart rate was calculated as the mean heart rate change from 0 to 8 seconds after onset. Post-stimulus inter-beat intervals were averaged across the first 2 inter-beat intervals and across intervals 3 to 5 (see Results for further details). Tukey post-hoc comparisons were used to determine changes in cardiac measures compared to baseline and differences between measures at different stimulus levels. Assumption of data normality was tested using residual normal probability plots. Statistical analyses were performed using Matlab 2016b (Mathworks, USA). A value of *p* <0.05 was considered statistically significant.

## Results

For all participants, hearing thresholds for ICRA noise were close to the lowest presentation level (the average threshold for participants was 10.2dB SPL, range 6–15.5 dB SPL). One participant had a threshold of 15.5 dB and the rest had thresholds between 6 and 13.5 dB. To obtain the ICRA hearing threshold (described in more detail in [5]), a 3 alternative forced choice method was used in which the turning point of perception was determined as the sound level at which 50% of the trials were heard and 50% missed. Therefore even with a hearing threshold of 15.5 dB, the participant would perceive some of the stimuli at 15 dB. Button presses at the offset of sound showed prompt responses for louder stimuli: however, at 15 dB SPL, some stimuli were missed (i.e. no button press) or delayed as participants either may not have perceived the stimulus, or were unsure when the stimulus had ended. At 15 dB SPL, on average 3.33 trials out of 10 (SD 3.17) were missed. Only one person (who had an ICRA threshold of 10 dB SPL) did not press the button within 4 seconds of the stimulus ending, for any of the 10 trials. (for button press time results see [5]).

### Heart rate change with intensity levels

**Fig 2:**
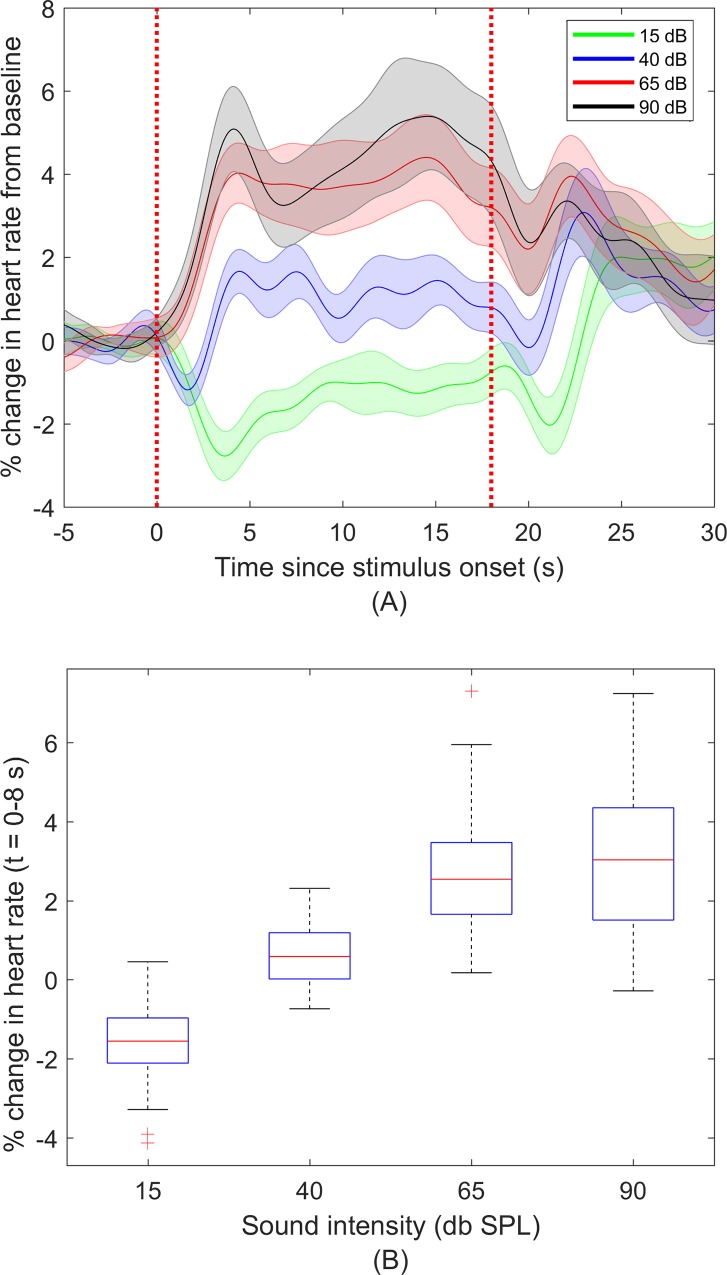
Percentage change in heart rate relative to baseline. (A) Percentage change in heart rate relative to baseline, averaged across participants (n = 27). Shaded areas show standard error of mean (SEM). (B) Mean heart rate change from t = 0 to 8 s relative to stimulus onset, averaged across participants. Boxes represent median, interquartile range and largest/ smallest non-outliers. Crosses represent outliers or values greater than 1.5 times the interquartile range. *** P<0.001.

Fig 2A shows the percentage change in heart rate following stimulus onset, averaged across participants. At 15 and 40 dB SPL, an initial drop and subsequent rise in heart rate was seen following stimulus onset. At 15 dB SPL (near the perceptual threshold for this sound for most participants) there was an average drop in heart rate of 2.8% at 3.6 s and at 40 dB SPL, an average drop of 1.1% at1.6 seconds was seen. At the higher intensity levels of 65 and 90 dB, average heart rate increased by approximately 4% and 5% respectively following stimulus onset.

At the higher stimulus levels of 65 and 90 dB a bi-phasic response with peaks at 4 and 14.5 seconds post-stimulus onset can be seen. At stimulus offset, an initial decrease in heart rate at all levels was observed following which heart rate at all stimulus levels approached baseline (Fig 2A).

To quantify this change in heart rate with sound onset, the mean heart rate change was calculated over the interval from 0 to 8 seconds post-stimulus onset (Fig 2B). This period was chosen to cover the first peak in heart rate change seen after sound onset in the grand averaged data. Heart rate changes were modelled using a linear mixed model. The change from baseline varied significantly between different stimulus levels (level x pre-post interaction) (*F*(3,182) = 59.77, *p* < .001). Multiple comparisons showed that compared to baseline, there was a significant drop in heart rate at 15 dB SPL (-1.55%, 95% CI -2.37 to -0.73%, *p* < .001) and a significant increase at 65 dB SPL (2.67%, 95% CI 1.85 to 3.50%, *p* < .001) and 90 dB SPL (3.0%, 95% CI 2.18 to 3.82%, *p* < .001). The change at 40 dB SPL over 8 seconds following stimulus onset was not significant. Comparing the post-stimulus changes in heart rate at different stimulus levels, a significant difference was found between all sound intensity levels (*p* < .001for all pairwise comparisons) except between 65dB and 90dB SPL (Fig 2B).

### Inter-beat intervals

**Fig 3:**
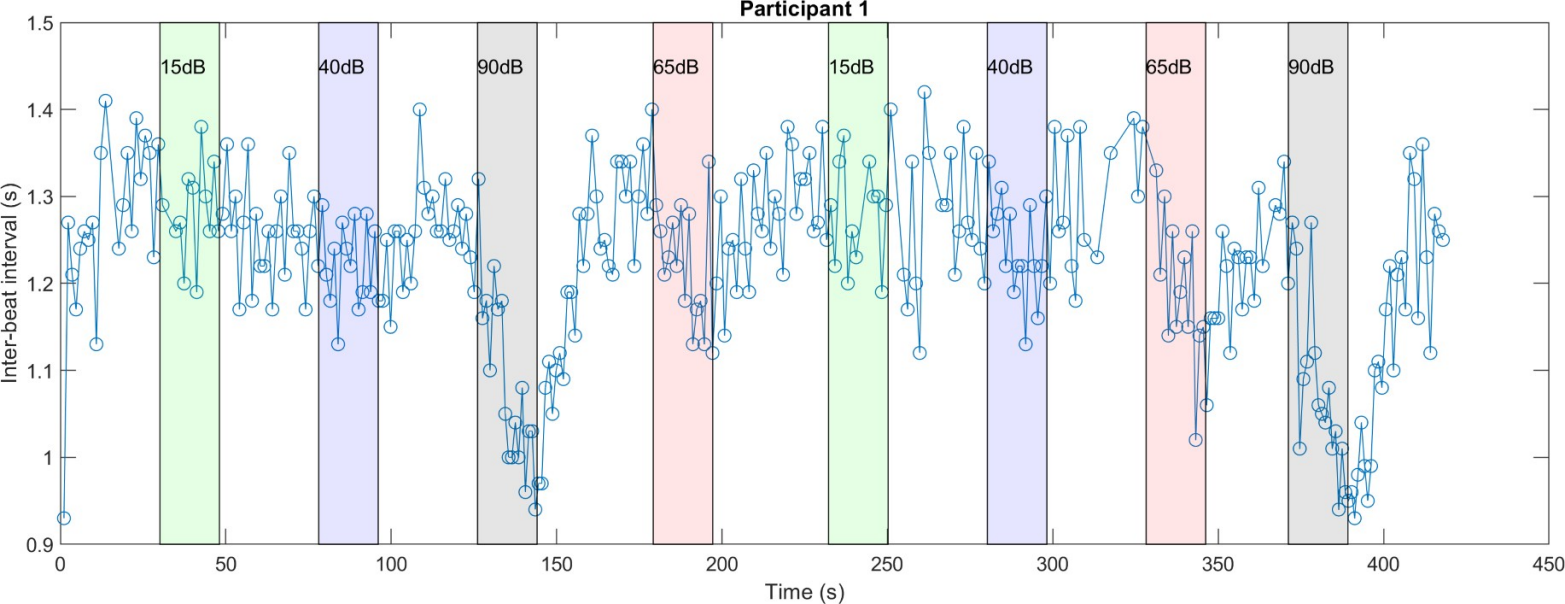
Inter-beat intervals across time for a representative participant. Vertical lines show the times sound stimuli at different levels were applied.

To quantify the time at which changes in heart rate first occurred, inter-beat intervals were assessed. Fig 3 shows inter-beat intervals for one participant during a seven minute recording. Shorter inter-beat intervals (corresponding to increased heart rate) following the 65 and 90dB SPL levels are clearly seen.

**Fig 4:**
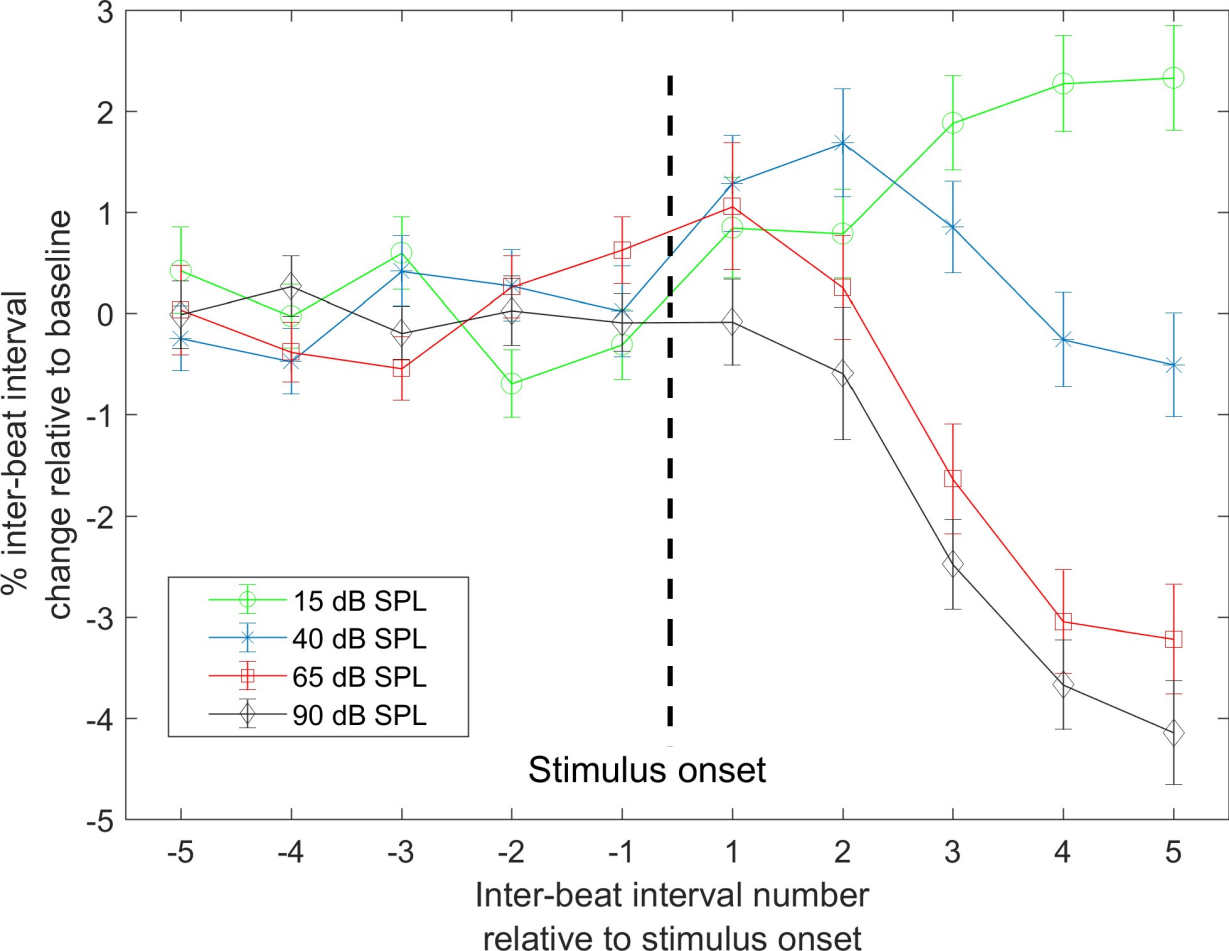
Percentage change in averaged inter-beat intervals relative to baseline. Values averaged across participants (n = 27) are shown. Error bars show standard error of mean (SEM).

To quantify the immediate change in inter-beat intervals following sound onset, the percentage change in post-stimulus inter-beat intervals relative to baseline (defined as the averaged five inter-beat intervals before stimulus onset), was calculated. Values averaged across all participants are shown in Fig 4. Post-stimulus inter-beat intervals were averaged across two ranges: 1) the first two intervals and 2) across intervals three to five. These choices were driven by the initial reduction and subsequent rise in average heart rate seen at the two lower stimulus levels (Fig 2A).

For each range of inter-beat intervals, a significant effect of stimulus level on the change in inter-beat interval relative to baseline (level x pre/post interaction) was found (post-stimulus averaged beats 1 and 2: *F*(3,182) = 3.16, *p* = 0.024, post-stimulus beats three to five: *F*(3,182) = 48.45, *p* < .001). Post-hoc comparisons showed that across the first two beats, the average percentage change in inter-beat intervals from baseline was only significant at 40 dB SPL (Table 1). At this sound level, the first two inter-beat intervals were significantly longer than baseline. Following the first two averaged inter-beat intervals at 40 dB SPL, values returned to baseline when averaged across intervals three to five (Fig 4). Averaged inter-beat intervals three to five were significantly longer than baseline at 15 dB SPL and significantly shorter at stimulus levels 65 and 90 dB SPL (Table 1). Following stimulus onset, the change in inter-beat intervals three to five from baseline was significantly different between all stimulus levels except 65and 90dB SPL (Table 2).

**Table 1:** Mean (95% confidence interval) percentage change in inter-beat intervals from baseline, across participants.

Stimulus level (dB SPL)	Mean (95% CI) inter-beat intervals 1 to 2 (% change from baseline)	*p* value	Mean (95% CI) inter-beat intervals 3 to 5 (% change from baseline)	*p* value
15	0.82 (-0.47 to 2.11)	0.529	2.17 (1.05 to 3.27)	<0.001***
40	1.48 (0.20–2.77)	0.011*	0.03 (-1.08 to 1.14)	1.0
65	0.66 (-0.62 to 1.94)	0.779	-2.63 (-3.75 to -1.52)	<0.001***
90	-0.34 (-1.62 to 0.94)	0.993	-3.43 (-4.54 to -2.32)	< 0.001***

* P<0.05

*** P<0.001

**Table 2:** Comparison between mean percentage change from baseline (95% confidence interval of difference) at different stimulus levels, across inter-beat intervals three to five.

Stimulus levels (dB SPL)	Difference of mean percentage change (95% CI)	*p* value
15–40	2.13 (1.01 to 3.25)	< .001
15–65	4.80 (3.68 to 5.91)	< .001
15–90	5.60 (4.48 to 6.71)	< .001
40–65	2.66 (1.55 to 3.78)	< .001
40–90	3.46 (2.35 to 4.58)	< .001
65–90	0.80 (-0.3 to 1.91)	0.371

## Discussion

Our results show that following onset of an ICRA noise stimulus and compared to baseline, heart rate initially decreased at the lower stimulus levels of 15 and 40 dB SPL, reaching a significantly lower rate at 15 dB SPL. At comfortably loud levels of 65 and 90 dB SPL increases in heart rate compared to baseline were seen. At stimulus offset, heart rates at all stimulus levels briefly decreased and subsequently returned to baseline. At the higher stimulus levels and across the duration of the stimulus, a biphasic response was seen which was more pronounced at 90 dB SPL.

To quantify the timing of changes, inter-beat intervals were assessed. At 40 dB SPL an immediate but short-duration change in inter-beat intervals was found whereas at other levels, the maximum change appeared later. Following stimuli onset, changes in inter-beat intervals were different among all levels except 65 and 90 dB SPL.

A number of early studies investigated changes in heart rate in response to various types of sound stimuli [9–13]. These studies mainly related changes to functional defensive and orienting responses [21]. Defensive responses occur to intense stimuli and are a protective reflex associated with the sensation of pain and decreasing sensitivity. The orienting response, which increases sensitivity during listening and facilitates reception of sensory information, has been shown to occur to any detectable change, including onset or termination of a stimulus [13, 21]. The orienting response has been described as intensified to stimuli near threshold or at high stimulus levels and weakened to stimuli of medium intensity [13, 22].

A review by Graham et al. suggested that heart rate deceleration with sound onset appeared to indicate an orienting response [14]. At 15 dB SPL (an intensity near the perceptual threshold), our results show a decrease in heart rate following stimulus onset that is consistent with this proposal. This change from baseline began immediately following sound onset and was statistically significant when averaged across inter-beat intervals three to five relative to stimulus onset. At 40 dB SPL (still a relatively low level), a brief but significant increase in averaged inter-beat intervals was seen before the intervals returned to baseline (Fig 4). This pattern is consistent with the orienting response being weakened at medium intensities [13, 22]. Our data also shows an initial decrease in heart rate following stimulus offset, which is also consistent with the orienting response occurring to termination of a stimulus. Decreases in heart rate at near-threshold levels could provide an indication of internal processing or attention to stimuli [23], in this case potentially showing that a near-threshold sound was perceived.

At the two higher stimulus levels we observed an increase in heart rate following stimulus onset. Different mechanisms for this increase have been suggested [12, 13]. Graham et al. suggested that an accelerating heart rate could be due to the defense response [14]. Roessler et al. suggested a behavioral startle response as a result of a short rise time in sound or due to high sound intensities [12]. However, the results of this study are not fully consistent with these proposals, since the stimuli used had an onset ramp and were not presented at a level high enough to induce a startle or defense response, especially at 65 dB SPL.

Increase in heart rate in response to sound has also been associated with respiratory changes [13]. Evidence for this stemmed from investigations of heart rate change during controlled and uncontrolled respiration which showed respiration increase was directly correlated with heart rate acceleration [24]. With sound applied [13], respiration amplitude showed significant increase suggesting higher heart rate was associated with respiration change. Since both of these changes are controlled by the autonomous nervous system, these results provide evidence supporting the notion that the onset of a sound activates the autonomous system differently depending on the level of the sound.

There have been reports of heart rate changes with sound which do not show the initial drop in heart rate at the lower levels that is seen in our data [12]. Roessler et al. recorded heart rate in response to five sound intensity levels between 40 and 120 dB SPL of a 2-second long 1 kHz tone [12]. Although these results did not show the initial reduction in heart rate, the authors concluded that the design of the experiment somewhat optimized a defense response of accelerating heart rate and minimized the orienting response. This was due partly to participants finding the higher intensities of stimulation distressing [12].

In our study, the overall changes in heart rate over the 18 second stimulus period at the two higher intensities and in particular at 90 dB SPL show a second peak or increase in heart rate around 14.5 seconds after stimulus onset (Fig 2). Previous studies which have looked at longer periods after sound onset have also reported this component [25–27]. Studies on effects of stimulus duration on heart rate have shown that the morphology of the heart rate response may depend on the duration of the stimulus; with stimuli of 15 seconds resulting in a biphasic response, similar to that observed in our study, but shorter 1- second stimuli resulting in a monophasic (i.e. acceleratory only) response [27]. The type and duration of the stimulus in this study were chosen in our original study [5] to ensure activation of a broad cortical region. Our results indicate that a shorter stimulus would generate a sound level dependent heart rate response and thus could be used in our future studies and in clinical applications.

Levels of drowsiness have been reported as a possible factor affecting heart rate changes to sound [28]. Although we did not include a measure of drowsiness in our study, participants were instructed to push a button at the end of each stimulus to help keep them focused during the recording. Quantifying levels of drowsiness however, may help with interpretation of further work.

In this study, to avoid any bias in selecting specific fNIRS channels from which to extract heart rate, we used an algorithm to exclude noisy channels and average the cardiac measures extracted from the remaining channels. For future development, only a single channel or a small number of channels would be sufficient for this application.

Our findings support previous data showing changes in cardiac activity in response to sound, and in addition characterize these changes using a broad range of sounds from near-threshold to comfortably loud levels. Cardiac measures can have clinical utility in objectively determining hearing thresholds and comfortable sound levels in infants or adults unable to provide behavioral feedback. Determining the dynamic range of hearing in this group of individuals can also assist in objectively programming hearing devices. The cardiac data in this study was extracted from fNIRS recordings of cortical activity to sound [5] and can therefore be used in combination with cortical measures to provide more accurate and objective assessments of hearing. A study by Schulman [7] showed a heart rate response to sound stimuli in infants with no auditory cortex, and for whom no cortical evoked potentials in response to sound were detected. This result shows the different neuroanatomical routes of cardiac and cortical responses and the potential to gain complementary information regarding hearing from each response.

## Supporting information

S1 Fig Percentage change in heart rate relative to baseline. (A) Percentage change in heart rate relative to baseline, averaged across participants (n = 27). The ‘resting’ heart rate trace shows the percentage change in eight seconds of resting data relative to the five seconds immediately before, showing inherent variability of the heart rate. Shaded areas show standard error of mean (SEM).
(TIF)

S1 Appendix Resting heart rate variability. (DOCX)

S1 Dataset Heart rate extracted from fNIRS recordings. (XLSX)

S2 Dataset Inter-beat intervals extracted from fNIRS recordings. (XLSX)
