# Supplementary figures and images for: Assessing hearing by measuring heartbeat: The effect of sound level
Source: PLoS One. 2019 Feb 28;14(2):e0212940. doi: 10.1371/journal.pone.0212940 (PMC6394942; doi:10.1371/journal.pone.0212940)

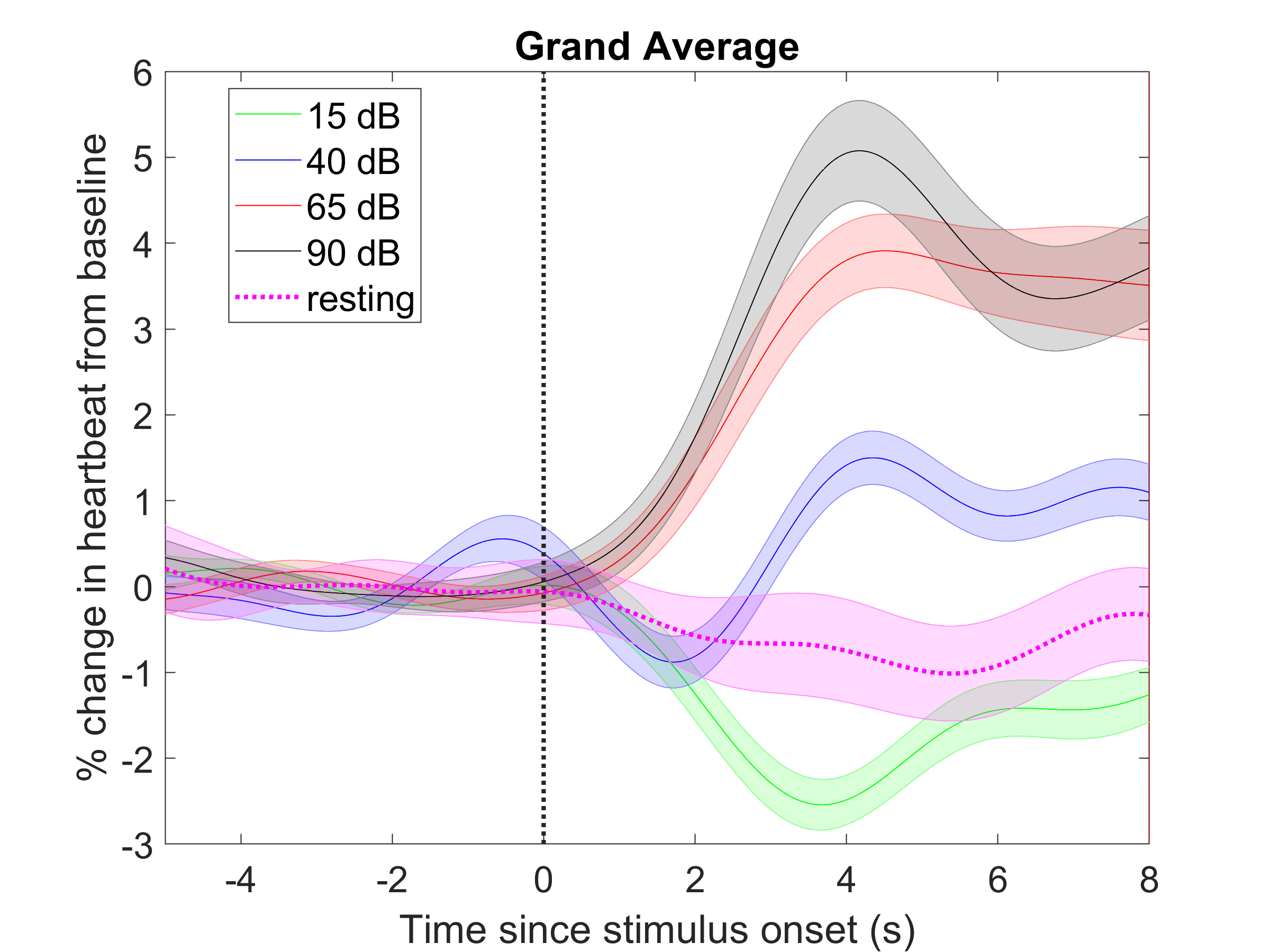

Supplement: S1 Fig — (A) Percentage change in heart rate relative to baseline, averaged across participants (n = 27). The ‘resting’ heart rate trace shows the percentage change in eight seconds of resting data relative to the five seconds immediately before, showing inherent variability of the heart rate. Shaded areas show standard error of mean (SEM). (TIF) [file pone.0212940.s001.tif]
