# Supplementary material for: Assessing hearing by measuring heartbeat: The effect of sound level
Source: PLoS One. 2019 Feb 28;14(2):e0212940. doi: 10.1371/journal.pone.0212940 (PMC6394942; doi:10.1371/journal.pone.0212940)
Supplement: S1 Appendix — (DOCX) [file pone.0212940.s002.docx]

# S1 Appendix - Resting Heart Rate Variability

To demonstrate variability of the resting heart rate and quantify the limit of the effect size measurable using this technique, we have used the heart rate from the resting periods recorded at the immediate start of each recording. A sham epoch was generated using the 13 seconds prior to the first stimulus; five seconds was used to simulate a baseline condition, followed by eight seconds to examine the heart rate variability. A period of eight seconds was chosen as it is comparable to the post-stimulus response over eight seconds, analyzed in the manuscript. Fig S1 shows the total 13 seconds plotted over the responses to sound levels. As seen, the post-stimulus change in heart rate in response to 15 and 40 dB does not overlap with the variability of resting heart rate.
